# Supplementary material for: Electromyographic analysis of stomatognathic muscles in elderly after hippotherapy
Source: PLoS One. 2020 Aug 27;15(8):e0238036. doi: 10.1371/journal.pone.0238036 (PMC7451555; doi:10.1371/journal.pone.0238036)
Supplement: Attachment — Submitted filename: Rebuttal Letter.docx [file pone.0238036.s001.docx]

Rebuttal Letter

Dear Professor Thomas A Stoffregen,

We were pleased to have an opportunity to revise our manuscript entitled "Electromyographic analysis of stomatognathic muscles in elderly individuals after hippotherapy." In the revised manuscript, we have carefully considered the editors' and reviewers' suggestions, and we adjusted some paragraphs accordingly. Below are the points we address and respond to each point raised by the editor and the reviewer. The responses to the editor's and reviewer's comments are below and are color-coded as follows: a) Comments from editors or reviewers are colored in red b) Our responses are shown under each comment as standard text. Overall, the comments were constructive, and we are appreciative of such constructive feedback on our original submission. After addressing the issues raised, we feel the quality of the paper is much improved.

Best regards,

Edneia Corrêa de Mello

Responses to editors’ comments:

In analyzing your results, you conducted many t-tests; at least 20. Did you make any correction for multiple comparisons, for example, in your criterion alpha? The submitted manuscript appears to suggest that you did not make such corrections. With appropriate correction for multiple comparisons, it is not clear that any of your effects would meet the criteria for statistical significance.

To avoid the type I error, common when many t-tests are conducted; we opted for Repeated Measured ANOVA with Bonferroni’s post hoc. We also chose to work with the electromyography data because much information is lost when normalizing the data (lines 152 - 161, on page 7).

1. Please ensure that your manuscript meets PLOS ONE's style requirements, including those for file naming. The PLOS ONE style templates can be found at

<https://journals.plos.org/plosone/s/file?id=wjVg/PLOSOne_formatting_sample_main_body.pdf> and

<https://journals.plos.org/plosone/s/file?id=ba62/PLOSOne_formatting_sample_title_authors_affiliations.pdf>

On the title page, we added the department and listed the corresponding author's initials in parentheses after the email address. We adjusted the names of the files. We hope that it now fits the style requirements, as described in the referred templates.

2. Please include your tables as part of your main manuscript and remove the individual files. Please note that supplementary tables should be uploaded as separate "supporting information" files.

We included our tables as part of the main manuscript and removed the individual files.

 3. Thank you for stating the following in the Acknowledgments Section of your manuscript:

"The authors are grateful for the financial support received for this research, provided by the following institutions: National Council for Scientific and Technological Development (CNPq), Coordination of Improvement of Higher-Level Personnel (CAPES), Foundation for Research Support of the State of Minas Gerais (FAPEMIG), Uberaba Teaching and Research Foundation (FUNEPU) and Association of Parents and Friends of the Exceptional of Uberaba (APAE)."

We note that you have provided funding information that is not currently declared in your Funding Statement. However, funding information should not appear in the Acknowledgments section or other areas of your manuscript. We will only publish funding information present in the Funding Statement section of the online submission form.

Please remove any funding-related text from the manuscript and let us know how you would like to update your Funding Statement. Currently, your Funding Statement reads as follows:

"No"

a. Please clarify the sources of funding (financial or material support) for your study. List the grants or organizations that supported your study, including funding received from your institution.
b. State what role the funders took in the study. If the funders had no role in your study, please state: “The funders had no role in study design, data collection and analysis, decision to publish, or preparation of the manuscript.”
c. If any authors received a salary from any of your funders, please state which authors and which funders.
d. If you did not receive any funding for this study, please state: “The authors received no specific funding for this work.”

Please include your amended statements within your cover letter; we will change the online submission form on your behalf

We removed all funding-related text from the manuscript and would like to keep our Funding Statement as we wrote in our Cover Letter.

Responses to reviewer #1:

There are several details that could be improved on this manuscript.
Overall organization of the manuscript is good but further improvements in this area will significantly improve clarity of the paper. It is striking that the Method section is on page 11. The results are found on page 4 of this paper. Does not agree with the guidelines of the magazine. Please check it. Thank you.

The Method section on page 11 was removed from this paper. “Non-habitual chewing is a standardized short-opening, flap-type, mouth-to-mouth movement required to reduce the effects of changing length and muscle tension typical of dynamic records. Thus, it eliminates some interference factors that act in chewing, such as swallowing between masticatory cycles, chewing frequency, food texture, and masticatory preference.”

The results found on page 4 were transferred to the Results section (lines 164 - 171, on page 6 and 7).

In addition to this, I include minor reorganization of a few parts of the manuscript, and significant rewriting of certain sections as suggested below:

1) INTRODUCTION: Line 68 to 71 on page 3: Please could you add the following reference in these lines?

Martín-Valero, R., Vega-Ballón, J., & Perez-Cabezas, V. (2018). Benefits of hippotherapy in children with cerebral palsy: A narrative review. European Journal of Paediatric Neurology, 22(6), 1150–1160. <http://doi.org/10.1016/j.ejpn.2018.07.002>

It is necessary to highlight the previously published results that found an increase in trunk strength and balance control. Hippotherapy is a treatment strategy that utilizes equine movement as part of a comprehensive program of intervention for the attainment of functional outcomes. It has been demonstrated that the recreational use of therapeutic riding can improve coordination, motor skills, posture and control of the head.

Thank you for the indication, we rewrote this paragraph and added the reference [13] (lines 61 – 71, on page 3).

2) Line 74 to 79 on Page 4. Please, I would recommend you that you can rewrite these phrases.

We rewrote these phrases (lines 74 – 78, on page 4).

MATERIAL AND METHODS:

3) Is the study registered in clinical trials with a reference number? Please, would you mind adding the reference number, Thank you in advance.

The study registered in clinical trials with this reference number [RBR-2kw6p9](http://www.ensaiosclinicos.gov.br/rg/RBR-2kw6p9/), and access is found on this link (<http://www.ensaiosclinicos.gov.br/rg/?q=RBR-2kw6p9>) (lines 86 and 87, on page 4).

4) Line 120 to 125 on page 5 and 6: Please could you add any reference in these lines?

We added references on these lines [17,18] [19] (lines 116 and 117, on page 5).

5) Rephrasing is needed in the following area to enhance clarity:
Line 127 to 141 on page 6, please, could you improve the wording in these lines? Please could you add any reference in these lines?

We rewrote this paragraph and added references [21] [22] (lines 119 - 136, on page 6 and 7).

6) DISCUSSION:
Line 235 to 242 on Page 10. Please, I would recommend you that you can rewrite these phrases.

We rewrote this paragraph (lines 250 - 255, on page 12).

7) CONCLUSION:
Line 260 to 262 on Page 11. Please, I would recommend you that you can rewrite these phrases.

We rewrote this paragraph (lines 270 and 271, on page 13).

Responses to reviewer #2:

I would like to suggest a modification in the title, in order that the readers can understand that this study is made with EMG.

We modified the title: Electromyographic analysis of stomatognathic muscles in elderly after hippotherapy

Thank you again for your time and effort,

Best regards

Edneia Mello
