# Supplementary material for: Electromyographic analysis of stomatognathic muscles in elderly after hippotherapy
Source: PLoS One. 2020 Aug 27;15(8):e0238036. doi: 10.1371/journal.pone.0238036 (PMC7451555; doi:10.1371/journal.pone.0238036)
Supplement: Attachment — Submitted filename: Response to Reviewers.docx [file pone.0238036.s002.docx]

Rebuttal Letter

Dear Professor Thomas A Stoffregen,

We were pleased to have the opportunity to revise our manuscript entitled "Electromyographic analysis of stomatognathic muscles in the elderly after hippotherapy" again. In the revised manuscript, we carefully considered Reviewer 1. We added more information about our statistical analysis. We explain the factors of the ANOVAs In the Section on Statistical Analysis. We have also included the F values and degrees of freedom associated with each p-value in Tables 1 and 2. We appreciate these constructive comments that will certainly change the way we conduct our future statistical analyzes.

Thank you again for your time and effort,

Best regards,

Edneia Corrêa de Mello
